# A Theoretical Study of the Interactions between Persistent Organic Pollutants and Graphene Oxide

**DOI:** 10.3390/ijerph191811340

**Published:** 2022-09-09

**Authors:** Qiuxuan Wu, Rui Zhang, Xiaoxiang Wang, Yizhuo Li

**Affiliations:** 1 School of Water Conservancy and Environment, University of Jinan, Jinan 250022, China; 2 Carbon Neutralization Technology Research Institute, Shenzhen Polytechnic, Shenzhen 518055, China; 3 Shenzhen Foreign Languages School, Shenzhen 518053, China

**Keywords:** persistent organic pollutants, graphene oxide, quantum chemical calculation, molecular doping, adsorption energy

## Abstract

Persistent organic pollutants (POPs) have adverse effects on the human health and ecosystem functioning. Graphene oxide (GO) has been developed to remove trace levels of POPs from wastewater samples. However, many questions involved in these processes are still unresolved (e.g., the role of π–π interaction, the effect of GO on the degradation of POPs, and so on). Revealing the microscopic interactions between GO and POPs is of benefit to resolve these questions. In the present study, a quantum chemical calculation was used to calculate the molecular doping and adsorption energy between eight representative POPs and GO. The influences of GO on the thermodynamic parameters, such as the Gibbs free energy and the highest occupied molecular orbital (HOMO)-lowest unoccupied molecular orbital (LUMO) gap, were also reported. We found the molecular doping is dependent on the species of POPs. The adsorption energy of the majority of POPs on GO is between 7 and 8 kJ/mol. Consequently, the GO may make degradation of POPs in wastewater more productive and lead to a change of kinetics of the degradation of POPs.

## 1. Introduction

Persistent organic pollutants (POPs) denote organic chemicals that are resistant to environmental degradation through photolytic, chemical, and biological processes [1]. They have half-lives ranging from decades to centuries in soil, water, and air [2,3]. Besides the persistence in the environment, compounds that make up POPs also have a high order of bioaccumulation and biomagnification. In detail, they can accumulate in the fatty tissue of living organisms including humans, and are found at higher concentrations at higher levels in the food chain [4,5]. They are also toxic to humans and wildlife, and are widely associated with adverse effects on growth, development, reproduction, and survival [6]. For example, polycyclic aromatic hydrocarbon (PAH), which is a kind of common POPs, is classified as possible or probable human carcinogens [7]. As an emerging POP of concern, perfluorooctane sulfonic acid (PFOS) has been found to be associated with fetal birth defects by altering the expression of crucial genes, reducing ATP production, inducing reactive oxygen species (ROS), and stimulating cell apoptosis in the early stages of cardiogenesis [8]. In addition, even the background-level exposure to polychlorinated biphenyls (PCBs) among children in the general population can also negatively influence their metabolic health [9]. The POPs are listed in Table 1, which were obtained from Stockholm Convention on Persistent Organic Pollutants [10]. The majority of these chemicals are currently or were in the past used as pesticides, solvents, and industrial chemicals. Many studies suggested that POPs can also be generated during the processes of burning events, industrial production, and urban garbage incineration [11].

Graphene oxide (GO), the oxidized analogue of graphene, is a two-dimension structured and oxygenated planar molecular material. It has been widely used in multidisciplinary fields, such as nonlinear optical materials [12,13], targeted drug carriers [14,15], and flexible rechargeable battery electrodes [16,17]. There are also many studies introducing GO into the advanced treatment of wastewater. Lee et al., demonstrated that the nanoplatelets of GO can suppress the fouling of membrane bioreactors during wastewater treatment [18]. Wu et al., used rhamnolipid-functionalized GO to adsorb methylene-blue from wastewater [19]. Zhao et al., suggested that few-layered GO nanosheets may be superior sorbents for heavy metals in ion pollution management [20]. Pavagadhi et al., found that GO could effectively adsorb microcystin-LR and microcystin-RR, and could be reused as an adsorbent following ten cycles of adsorption/desorption with no significant loss in its adsorption capacity [21]. Piaskowski et al., revealed that GO materials could affect physicochemical and biological processes of wastewater treatment [22]. Actually, GO naturally have significant pore volume, high conductivity, and large surface area. These physicochemical properties make it have excellent adsorption and catalytic capacity on organic pollutants, which can be used to remove organic pollutants in the aqueous phase [23]. The number of studies involved in GO for the removal of trace levels of POPs from water samples also keeps increasing in the past decade. In Zeng et al.’s study, the synthesized Fe_3_O_4_@GO presented a high enrichment of PCBs from environmental water samples [24]. Le et al. introduced GO into the electron-Fenton process to remove dye pollutants, presenting quite high efficiency [25]. Koushik et al. developed a method to perform rapid dehalogenation by using GO–silver nanocomposite [26]. Gupta et al. utilized a superhydrophobic sponge coated with reduced GO to separate oils and organic solvents from water [27]. Uddin et al., demonstrated that reduced GO-NiFe_2_O_4_ nanocomposites could be used for the electrochemical oxidation of hydrazine [28]. Wu et al., found that reduced GO-ferrite hybrids were effective adsorbents for sulfonamides in wastewater [29].

Although the processes of removing different kinds of POPs by GO were studied profoundly, many questions involved in these processes are still unresolved. For example, the roles of π–π interaction, the effects of GO on the degradation of POPs, etc. Revealing the interactions between GO and POPs is useful not only to understand the influences of GO on POPs, but also to promote the modification of GO for better treatment of POPs in wastewater. In the present study, a semi-empirical quantum chemistry method, AM1, was used to calculate the molecular doping and adsorption energy between eight selected POPs and GO. The influence of GO on the thermodynamic parameters, such as the Gibbs free energy, was also reported. Furthermore, physical-chemistry models were implemented with the data from quantum chemical calculation to illustrate the potential significance of GO for removal of POPs in wastewater treatment.

## 2. Methods

### 2.1. Chemicals

**Figure 1:**
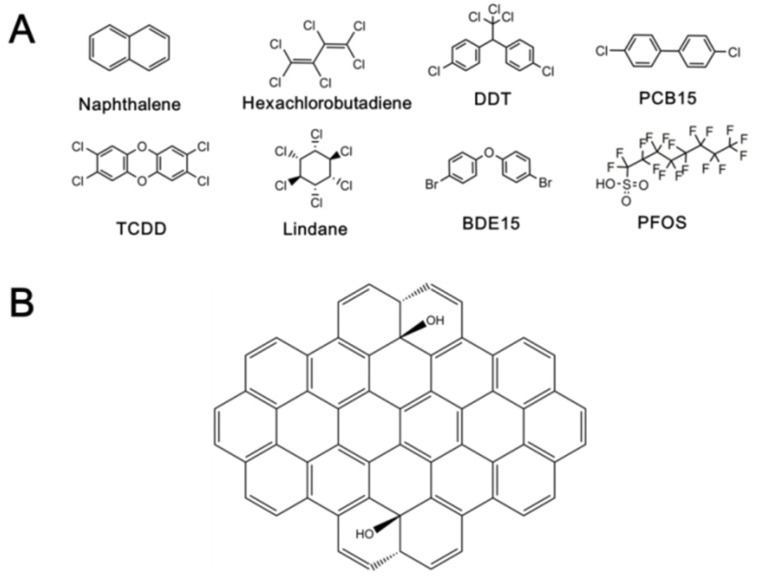
The structure of studied chemicals (**A**) and the proxy of GO (**B**).

Several typical chemicals, naphthalene, hexachlorobutadiene, dichloro-diphenyl-trichloroethane (DDT), PCB15, 2,3,7,8-tetrachlorodibenzo-*p*-dioxin (TCDD), lindane, 4,4′-dibromodiphenyl ether (BDE15), and PFOS, as the representative of POPs were studied here. The information about these chemicals is listed in Table 2, and the structures are shown in Figure 1A. In the Stockholm Convention on Persistent Organic Pollutants (POPs), POPs are divided into three categories: pesticides, industrial chemicals, and unintentional productions. Eight ones commonly found in the environment were selected to cover the three categories for the present study. For PCDDs, PCBs, and PBDEs, which contain a variety of congeners, we selected the most representative ones, i.e., TCDD, PCB15, and BDE15, respectively. Therefore, the results based on these chemicals are also informative for the studies of other different POPs. The GO supercell (58 carbon atoms, Figure 1B) was selected as the proxy of GO.

### 2.2. Computational Details

**Figure 2:**
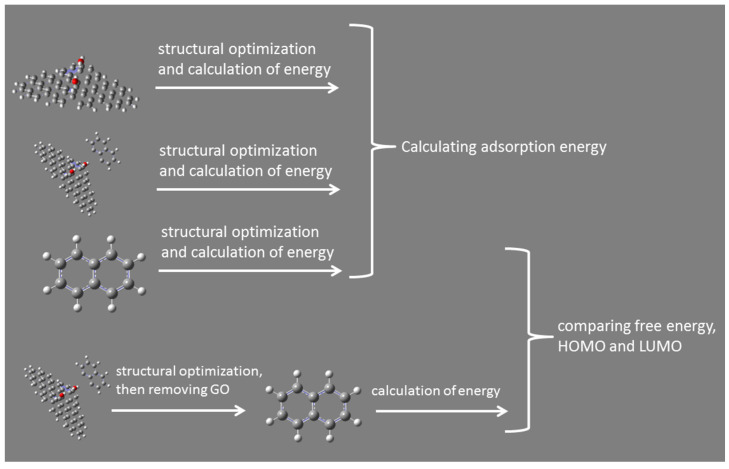
The flow chart of calculation in this study.

All of the optimization and vibrational frequencies analysis mentioned below were performed using Gaussian 09 (Gaussian Inc., Wallingford, CT, USA) with AM1 method. As a computational physics and chemistry program, Gaussian 09 software typically uses density functional theory (DFT) method for electronic and geometric structure optimization (geometric optimization, transition states, single-point calculation, and reaction path modeling), molecular properties, vibration analysis, electrostatic potential, electron density, and multipole moments. However, given that the systems studied here were a bit large, the AM1 method was used to facilitate the investigation of molecular doping and adsorption energy. The schematic of all the procedures was summarized in Figure 2, and naphthalene was presented as a proxy of all studied POPs. To evaluate the electron transfer between POPs and GO, the structures of complex of each POPs and GO were optimized. The process of finding the minimum energy configuration of molecules through geometric optimization was accomplished by locating the minimum and transition states on the potential plane of molecular orbitals. Gaussian 09 calculated the wave function and energy of the initial geometry and then went on to find new geometries with lower energy. The lowest energy geometry was found by repeating this process. Mulliken atomic charges of each atom were obtained by vibrational frequencies analysis. The charge distribution on atoms indicated the formation of donor and acceptor pairs involved in charge transfer in molecules. Mulliken atomic charge affected dipole moment, molecular polarization, electronic structure and other properties of molecular systems, and played an important role in quantum mechanical calculations [30]. The total number of changes on the atoms of POPs is the number of changes of the corresponding molecules, and then we can identify whether the molecules get or lose electrons. The adsorption energy of different POPs on the surface of GO is calculated by Equation (1),

(1) $$E_{adsorption}=E_{tot}\left(GO+POPs\right)-E_{tot}\left(GO\right)-E_{tot}\left(POPs\right),$$

where $E_{adsorption}$ is the adsorption energy, which was used to reveal whether GO can eliminate POPs and the related removal mechanisms, $E_{tot}\left(GO+POPs\right)$ is total energy of complex of GO and POPs, $E_{tot}\left(GO\right)$ is total energy of GO and $E_{tot}\left(POPs\right)$ is total energy of each studied chemicals. GO also affects the Gibbs free energy, HOMO and LUMO. These quantum chemical parameters are informative for the application of GO for eliminating POPs from wastewater.

The values of isolated POPs were calculated and compared to the values of POPs undergoing the influence of GO. To calculate values for isolated chemicals, it is needed to optimize the structure of studied chemicals individually and to analyze the respective vibrational frequencies. To get the values influenced by GO, we optimized the structure of studied chemicals with GO first, and then removed GO and performed vibrational frequency analysis on the corresponding studied chemicals.

## 3. Results and Discussion

### 3.1. Molecular Doping on GO and Adsorption Energy

Molecular doping, which means that charge transfer between the studied POPs and the surface of GO, is analyzed. As shown in Table 3, naphthalene, DDT, TCDD, and lindane are electron acceptors, and they get 0.00048, 0.00113, 0.000698, and 0.000977 charges from GO, respectively. Hexachlorobutadiene, PCB15, BDE15, and PFOS are electron donors, and they give 0.00134, 0.003182, 0.002278, and 0.002611 charges to GO, respectively. When the organic chemical is the electron donor, the number of electron transfer should be many times higher than when it is the electron acceptor, which may be caused by the fact that electrons on GO have much stronger delocalization potential than those on organic molecules. Therefore, as long as GO can absorb electrons, it tends to maintain the state of absorbing electrons all the time. By contrast, if a small organic molecule absorbs electrons, those electrons are immediately close to “saturation”. Leenaerts et al., have studied molecular doping of small inorganic molecules (e.g., H_2_O and NH_3_) adsorbed on the surface of graphene [31]. The number of electron transfer from their study is 1–2 orders of magnitude larger than that obtained here. This difference can be attributed to the difference of adsorbent (graphene vs GO) or adsorbate (small inorganic molecules vs POPs). Further research on HOMO and LUMO values when GO is present or absent with the solvation effect would be helpful in understanding this difference.

$E_{adsorption}$ of these 8 POPs were calculated and listed in Table 3. Seven of these values are between 7 and 8 kJ/mol. It implies the majority of POPs have a similar value of adsorption energy on GO, and more experimental research are needed certainly to validate this theoretical inference. However, the one for TCDD is only ~2.8 kJ/mol, which means the interaction between TCDD and GO is much weaker than that for other kinds of POPs. It may be caused by the π–π interaction [32] between TCDD and GO. Xiao et al., found that π–π electron-donor-acceptor interaction (EDA) interaction was not conducive to the delocalization of positively charged alkyl groups and charges into other rings, and the charge was far beyond the correlation with ring insulation amines [33]. The relationship between molecular doping and adsorption energy was also analyzed, and no significant relationship was found. Therefore, the results obtained by the AM1 method indicated that the adsorption of the POPs studied here on GO may be independent on molecular doping. As only eight chemicals were studied here, the robustness should be validated by more cases. However, Yang et al., investigated the adsorption of 1-naphthylamine, 1-naphthol, and naphthalene by GO/iron oxide composites, and they revealed that the adsorption of aromatic compounds was primarily influenced by EDA interaction [34]. The different views on adsorption mechanism may be due to the different adsorbents and compounds used.

### 3.2. Potential Influence on Degradation Reactions

The effect of GO on the degradations of POPs in wastewater is analyzed. The reaction of degradation can be presented by

(2) POPs⇄products,

The reaction gets equilibrium when Equation (3) occurs.

(3) $$G_{POPs}=G_{product},$$

**Figure 3:**
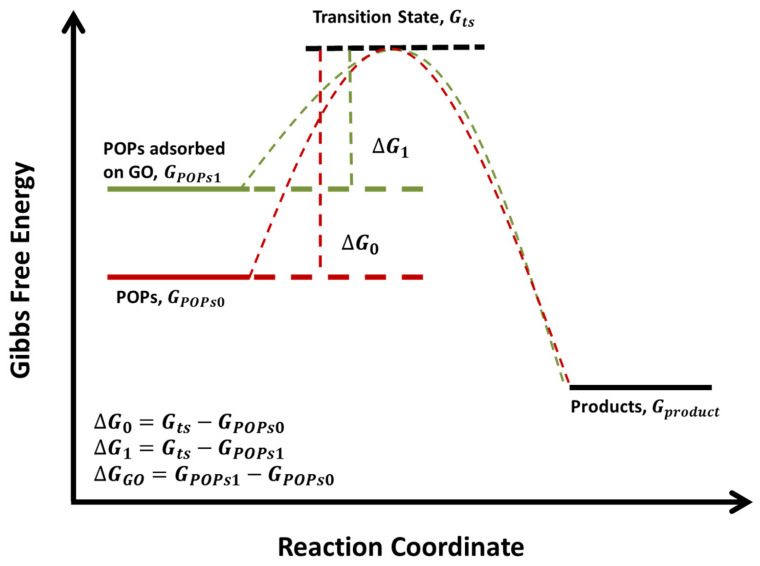
The conceptual diagram of the Gibbs free energy profile versus the reaction coordinate.

Here, $G_{POPs}$ is Gibbs free energy of POPs as mentioned above. The value is $G_{POPs0}$ when the chemical is isolated, and the value is $G_{POPs1}$ when the chemical is adsorbed onto the surface of GO. $G_{product}$ is sum of Gibbs free energy of all kinds of products, and its value is assumed not to be affected by GO. Before equilibrium, all reactants will experience transition state. By following these basic concepts, a diagram of the Gibbs free energy profile plotted versus the reaction coordinate is made (Figure 3). $G_{ts}$ is the Gibbs free energy of POPs at transition state, $\Delta G_{0}$ (=$G_{ts}-G_{POPs0}$) and $\Delta G_{1}$ (=$G_{ts}-G_{POPs1}$) are the values of energy barrier. $\Delta G_{GO}$ denotes the difference between $G_{POPs1}$ and $G_{POPs0}$ ($\Delta G_{GO}=G_{POPs1}-G_{POPs0}$). $G_{POPs0}$ and $G_{POPs1}$ of each chemical were calculated (Table 4). $G_{POPs1}$ is systematically larger than $G_{POPs0}$, demonstrating that GO may affect the thermodynamic equilibrium of the reactions involved in the degradation. $\Delta G_{GO}$ of DDT and TCDD are the largest ones, it may be attributed to the interaction between GO and multiple chlorine atoms of these chemicals, which is supported by the finding that GO is capable of adsorbing chlorine atoms [35].

GO can decrease the energy barrier of reactions involved in degradation. Thus, no matter what is the value of $G_{ts}$, we can always get $\Delta G_{0}>\Delta G_{1}$. It can be explained by the following equation.

(4) $$\Delta G_{0}-\Delta G_{1}=\left(G_{ts}-G_{POPs0}\right)-\left(G_{ts}-G_{POPs1}\right)=G_{POPs1}-G_{POPs0}=\Delta G_{GO}>0,$$

$\Delta G_{GO}$ of all chemicals are positive as shown in Table 4. A smaller energy barrier means that reactions can occur more easily. In other words, more POPs can be degraded when GO is present. This point can be further elucidated by analyzing the change of equilibrium constant (K) which can be written as the following equation.

(5) $$K=\frac{{\left[Product_{1}\right]}^{b1}\cdot {\left[Product_{2}\right]}^{b2}\ldots \ldots {\left[Product_{n}\right]}^{bn}}{{\left[\mathrm{POP}\right]}^{a1}},$$

where [X] represents the concentration of X. a and b are the stoichiometry of reactants and products, respectively. From the definition of equilibrium constant from the perspective of statistical physics, K can be written as follows:

(6) $$K=\exp\left(\frac{-G_{p-r}}{RT}\right),$$

$G_{p-r}$ denotes the difference between the Gibbs free energy of products and reactants, here it is the value of $G_{product}-G_{POPs0}$ or $G_{product}-G_{POPs1}$. R is the gas constant, and T is the temperature in Kelvin. The exact value of $G_{product}$ for each chemical cannot be calculated because the final products of these chemicals are unknown. However, no matter what values $G_{product}$ is, the value of $\exp\left(\frac{-\left(G_{product}-G_{POPs1}\right)}{RT}\right)$ is always greater than $\exp\left(\frac{-\left(G_{product}-G_{Pops0}\right)}{RT}\right)$ because

(7) $$-\left(G_{product}-G_{POPs1}\right)-\left[-\left(G_{product}-G_{POPs0}\right)\right]=G_{POPs1}-G_{POPs0}=\Delta G_{GO}>0,$$

Therefore, the equilibrium constant without the influence of GO is larger than that when GO is absent.

### 3.3. Changes of HOMO and LUMO

The effects of GO on the HOMO and LUMO of POPs are studied in this section. In Table 5, all values of HOMO and LUMO when GO is present (HOMO_1_ and LUMO_1_) or absent (HOMO_0_ and LUMO_0_) are shown. According to the frontier molecular orbital theory [36], HOMO-LUMO gap ($\Delta E_{HOMO-LUMO}$), which denotes the energy difference between the HOMO and LUMO, can be used to describe the stability of chemicals. So, this value was also calculated (Table 5). HOMO is the outermost orbital that contains the electron and can act as an electron donor orbital. The LUMO is the innermost orbital that has space to accept electrons which can act as an electron acceptor [30]. The difference in energy between the HOMO and the LUMO is an important indicator of stability. The larger the value of $\Delta E_{HOMO-LUMO}$, the most stable the molecule is in chemical reactions.

The HOMO-LUMO gap of several compounds with or without GO were compared. The results showed that the order of $\Delta E_{HOMO-LUMO}$ is lindane > PFOS > hexachlorobutadiene > DDT > BDE15 > PCB15 > naphthalene > TCDD. The addition of GO did not affect the overall ordering of $\Delta E_{HOMO-LUMO}$ for the compounds studied. But it did cause a change in the HOMO–LUMO energy gap. $\Delta E_{HOMO-LUMO}$ of naphthalene, DDT, TCDD, and BDE15 are increased by GO, while the values of hexachlorobutadiene, PCB15, lindane, and PFOS are decreased. These changes may affect the kinetics of the degradation of POPs. For example, in sulfate radical-advanced oxidation processes (SR-AOPs) of wastewater [37,38,39], the relationship between the rate constants ($k_{SO_{4}^{.-}}$) of reaction and ΔE can be described by Equation (8) [40].

(8) $$k_{SO_{4}^{.-}}=26.8-3.97\times \#O:C-0.746\times \Delta E_{HOMO-LUMO},$$

where #O:C is the ratio of number of oxygen to carbon atoms. For one chemical, #O:C keeps unchanging, so Equation (8) suggests that $k_{SO_{4}^{.-}}$ becomes smaller if $\Delta E_{HOMO-LUMO}$ increases, and larger if $\Delta E_{HOMO-LUMO}$ decreases.

On the other hand, the present results also provide ideas for the chemical removal of POPs. GO changes the $\Delta E_{HOMO-LUMO}$ of POPs, and it may lead to a change of kinetics of the chemical degradation of POPs. In the next study, the effects of GO on the chemical degradation of POPs, the relationships between the degradation kinetics of POPs and the $\Delta E_{HOMO-LUMO}$ as influenced by GO, etc., should be further investigated by combining experiments and theoretical calculations, such as DFT method and thermal degradation kinetics experiment.

## 4. Conclusions

In summary, we used a quantum chemical calculation to illustrate the interaction between GO and different kinds of POPs. The molecular doping is dependent on the POPs and no unified law applies to these chemicals. The adsorption energy of POPs on GO is calculated, and the majority of these values are between 7 and 8 kJ/mol. The GO increases Gibbs free energy, and it implies GO can make degradation of POPs in wastewater more productive. GO also changes $\Delta E_{HOMO-LUMO}$ of POPs, and it may lead to a change in kinetics of the degradation of POPs. However, more sets of POPs are needed to be measured theoretically and experimentally to validate the conclusions. In addition, the present work demonstrates that the innovative method, quantum chemical calculation complemented with thermodynamic and kinetic analyses, introduced in this work can be used to facilitate the study of the interactions between organic pollutants and GO during the process of wastewater treatment.

## Figures and Tables

**Table 1:** All POPs listed in the Stockholm Convention.

POPs	Treatment	Function
Aldrin	E	P
Chlordane	E	P
Chlordecone	E	P
Decabromodiphenyl ether	E	I
Dicofol	E	P
Dieldrin	E	P
Endrin	E	P
Heptachlor	E	P
Hexabromobiphenyl	E	I
Hexabromocyclododecane (HBCDD)	E	I
Hexabromodiphenyl ether and heptabromodiphenyl ether	E	I
Hexachlorobenzene (HCB)	E & RU	P & I & U
Hexachlorobutadiene	E	I
Alpha hexachlorocyclohexane	E	P
Beta hexachlorocyclohexane	E	P
Lindane	E	P
Mirex	E	P
Perfluorooctanoic acid (PFOA), its salts and PFOA-related compounds	E	I
Pentachlorobenzene	E & RU	P & I & U
Pentachlorophenol and its salts and esters	E	P
Polychlorinated biphenyls (PCBs)	E & RU	I & U
Polychlorinated naphthalenes (PCNs)	E & RU	I & U
Short-chain chlorinated paraffins (SCCPs)	E	I
Technical endosulfan and its related isomers	E	P
Tetrabromodiphenyl ether and pentabromodiphenyl ether	E	I
Toxaphene	E	P
Dichloro-diphenyl-trichloroethane (DDT)	R	P
Perfluorooctane sulfonic acid (PFOS), its salts and perfluorooctane sulfonyl fluoride	R	P & I
Hexachlorobutadiene (HCBD)	RU	U
Polychlorinated dibenzo-p-dioxins (PCDD)	RU	U
Polychlorinated dibenzofurans (PCDF)	RU	U

E: elimination, R: restriction, RU: reduce the unintentional releases, P: pesticide, I: industrial chemical, U: unintentional production.

**Table 2:** The information about studied chemicals.

Chemicals	Abbreviation	CAS Number
Naphthalene	-	91-20-3
Hexachlorobutadiene	-	87-68-3
Dichloro-diphenyl-trichloroethane	DDT	50-29-3
4,4’-Dichlorobiphenyl	PCB15	2050-68-2
2,3,7,8-Tetrachlorodibenzo-*p*-dioxin	TCDD	1746-01-6
Lindane	-	58-89-9
4,4’-Dibromodiphenyl ether	BDE15	2050-47-7
Perfluorooctanesulfonic acid	PFOS	1763-23-1

**Table 3:** Molecular doping and adsorption energy of POPs.

Chemicals	Charge (e)	$E_{adsorption}\;(\mathrm{kJ}/\mathrm{mol})$
Naphthalene	−0.00048	7.393128
Hexachlorobutadiene	0.00134	7.44752
DDT	−0.001138	7.577224
PCB15	0.003182	7.619064
TCDD	−0.000698	2.878592
Lindane	−0.000977	7.401496
BDE15	0.002278	7.694376
PFOS	0.002611	7.669272

**Table 4:** Gibbs free energy of POPs (Hartree).

Chemicals	$G_{POPs0}$	$G_{POPs1}$	$\Delta G_{GO}$
Naphthalene	0.184766	0.184823	0.000057
Hexachlorobutadiene	−0.02017	−0.020166	0.000004
DDT	0.177261	0.177815	0.000554
PCB15	0.181256	0.181503	0.000247
TCDD	0.061355	0.061894	0.000539
Lindane	−0.02659	−0.026539	0.000051
BDE15	0.179335	0.179559	0.000224
PFOS	−1.35958	−1.35956	0.00002

**Table 5:** HOMO, LUMO, and the corresponding gap of POPs (Hartree).

Chemicals	HOMO_0_	LUMO_0_	$\Delta E_{0\_HOMO-LUMO}$	HOMO_1_	LUMO_1_	$\Delta E_{1\_HOMO-LUMO}$
Naphthalene	−0.32014	−0.00973	0.31041	−0.3201	−0.00968	0.31042
Hexachlorobutadiene	−0.36912	−0.02335	0.34577	−0.36903	−0.02346	0.34557
DDT	−0.3523	−0.01901	0.33329	−0.35288	−0.01936	0.33352
PCB15	−0.33307	−0.01991	0.31316	−0.33244	−0.0205	0.31194
TCDD	−0.33065	−0.03342	0.29723	−0.3314	−0.03329	0.29811
Lindane	−0.41761	−0.00552	0.41209	−0.41754	−0.00588	0.41166
BDE15	−0.33709	−0.01477	0.32232	−0.33766	−0.01503	0.32263
PFOS	−0.43885	−0.08665	0.3522	−0.43828	−0.08679	0.35149

## Data Availability

All data generated or analyzed during this study are included in this published article.

## Abbreviations

The following abbreviations are used in this manuscript:

POPs	Persistent organic pollutants
GO	Graphene oxide
HOMO	Highest occupied molecular orbital
LUMO	Lowest unoccupied molecular orbital
PAH	Polycyclic aromatic hydrocarbon
PFOS	Perfluorooctane sulfonic acid
ROS	Reactive oxygen species
PCBs	Polychlorinated biphenyls
DDT	dichloro-diphenyl-trichloroethane
TCDD	2,3,7,8-tetrachlorodibenzo-*p*-dioxin
BDE15	4,4′-dibromodiphenyl ether
EDA	Electron-donor-acceptor
SR-AOPs	Sulfate radical-advanced oxidation processes
